# Supplementary material for: Snow Albedo Seasonality and Trend from MODIS Sensor and Ground Data at Johnsons Glacier, Livingston Island, Maritime Antarctica
Source: Sensors (Basel). 2019 Aug 15;19(16):3569. doi: 10.3390/s19163569 (PMC6720592; doi:10.3390/s19163569)
Supplement: Supplementary file 1 [file sensors-19-03569-s001.pdf]

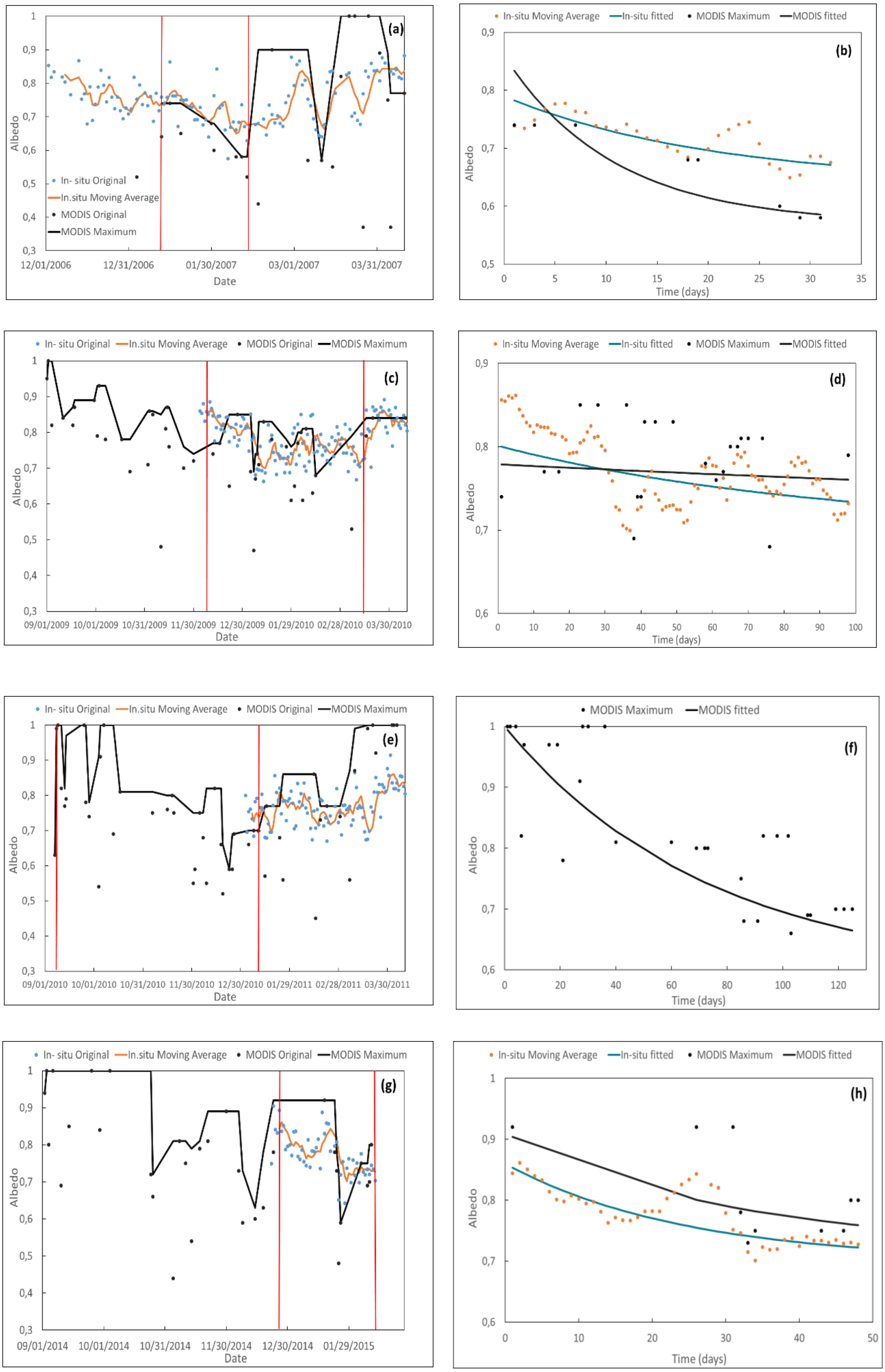

Figure S1: Left column: original in-situ albedo (blue dots), original MOD10A1 albedo (black dots), in-situ albedo moving average (brown solid line) and filtered MOD10A1 albedo (black solid line) from 1

September to 10 April. Right column: in-situ albedo moving average (brown dots) and filtered MOD10A1 albedo and the corresponding fit to the exponential decay. Season 2006–2007 ((**a**) and (**b**)), season 2009–2010 ((**c**) and (**d**)), season 2010–2011 ((**e**) and (**f**)), season 2014–2015 ((**g**) and (**h**)). The vertical red lines in (**a**), (**c**), (**e**) and (**g**) indicate the start and end dates of the snow melting.

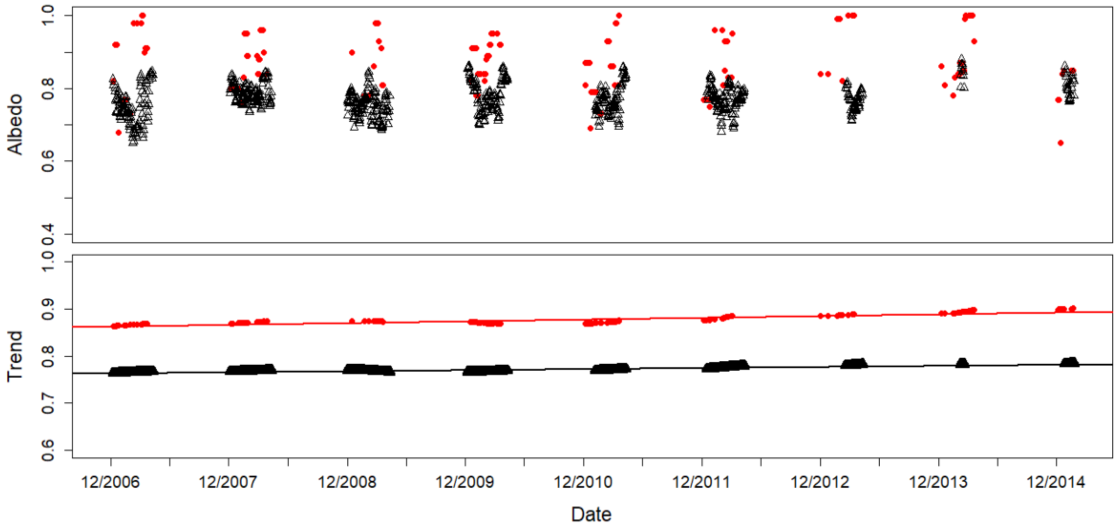

Figure S2: MOD10A1 version 6 and in-situ data trend analysis. Moving average of in-situ albedo (open triangles) and maximum values of MOD10A1 albedo (red dots) from 1 December to 10 April (upper figure) and the calculated trends of in-situ moving average (open triangles) and MOD10A1 maximum albedo (red dots) from 1 December to 10 April (bottom figure). The linear fits of the trend are also shown.
